# RNA-Seq Analysis Identifies Differentially Expressed Genes in Subcutaneous Adipose Tissue in Qaidaford Cattle, Cattle-Yak, and Angus Cattle

**DOI:** 10.3390/ani9121077

**Published:** 2019-12-03

**Authors:** Chengchuang Song, Yongzhen Huang, Zhaoxin Yang, Yulin Ma, Buren Chaogetu, Zhaxi Zhuoma, Hong Chen

**Affiliations:** 1 College of Animal Science and Technology, Northwest A&F University, Yangling 712100, China; chengchuangsong@163.com (C.S.); hyzsci@nwafu.edu.cn (Y.H.); yangzhaoxin1994@163.com (Z.Y.); 2 Animal Disease Control Center of Haixi Mongolian and Tibetan Autonomous Prefecture, Delingha 817000, China; mylqinghai@163.com (Y.M.); dong_ongd@163.com (B.C.); yunma_nux@163.com (Z.Z.)

**Keywords:** cattle-yak, subcutaneous fat, transcriptome analysis, meat quality

## Abstract

**Simple Summary:**

Fat content is an important factor affecting beef quality. Therefore, the screening and identification of differentially expressed genes in adipose tissue between different breeds (Qaidamford cattle, hybrid cattle-yak, and Angus cattle) by RNA high-throughput sequencing can provide useful information to the beef cattle industry. The aim of this work was to identify candidate genes of adipose tissue for future beef breeding efforts. Comparative analysis revealed a significant difference between hybrid cattle and Angus, but the difference between hybrid cattle varieties (cattle-yak vs. Qaidamford cattle) was not significant. Gene ontology (GO) and KEGG pathway enrichment analysis indicated that some differentially expressed genes are involved in lipid metabolism-related biological processes and signaling pathways associated with cell metabolism, such as extracellular matrix (ECM)-receptor interaction and the PI3K-Akt signal pathway. The expression levels of some of the identified genes were further verified by reverse transcription quantitative polymerase chain reaction (RT-qPCR). These data will be helpful for further investigations of meat quality and breeding efforts for different cattle breeds.

**Abstract:**

In the beef industry, fat tissue is closely related to meat quality. In this study, high-throughput RNA sequencing was utilized for adipose tissue transcriptome analysis between cattle-yak, Qaidamford cattle, and Angus cattle. The screening and identification of differentially expressed genes (DEGs) between different breeds of cattle would facilitate cattle breeding. Compared to Angus cattle adipose tissue, a total of 4167 DEGs were identified in cattle-yak adipose tissue and 3269 DEGs were identified in Qaidamford cattle adipose tissue. Considering cattle-yak as a control group, 154 DEGs were identified in Qaidamford cattle adipose tissue. GO analysis indicated the significant enrichment of some DEGs related to lipid metabolism. The KEGG pathway database was also used to map DEGs and revealed that most annotated genes were involved in ECM-receptor interaction and the PI3K-Akt signal pathway, which are closely related to cell metabolism. Eight selected DEGs related to adipose tissue development or metabolism were verified by RT-qPCR, indicating the reliability of the RNA-seq data. The results of this comparative transcriptome analysis of adipose tissue and screening DEGs suggest several candidates for further investigations of meat quality in different cattle breeds.

## 1. Introduction

Qaidamford cattle are ternary hybrid cattle with good meat quality [1]. Qaidamford cattle were bred using Y*ak*, Qaidam cattle, and imported Angus cattle, and then reproduced by hybridization technology. Qaidamford cattle have several advantages, including fast growth, high yield, good meat quality, and strong adaptability [1]. Among these excellent traits, meat quality is essential for economic value in the beef cattle industry. Many factors affect meat quality traits, such as softness, hardness, oxidation stability, color, flavor, muscle fiber, fat composition, and fat content [2,3]. Meat quality assessment found a significantly higher ability of Qaidamford cattle to deposit fat compared to that of yak, resulting in improved meat quality [1]. Thus, it is of great significance to analyze meat quality traits by comparing fat tissue transcriptome data for different varieties of cattle.

Large amounts of fat deposits in adipose tissue are closely related to obesity and energy metabolism abnormalities, and can contribute to disorders like Type 2 diabetes, insulin resistance, and cardiovascular disease in humans [4]. Numerous studies have shown that fat deposition in animals is a complex biological process that includes regulation by multiple transcriptional factors, such as PPARγ, some members of the C/EBP family, KLFs, STAT5 [5], SREBP-1c [6,7], the E2F family of transcription factors, and Wnts. Thus, the screening of differentially expressed genes between different bovine fat tissues can facilitate further study of fat deposition. The goal of this work was to investigate differences in the subcutaneous fat transcriptome between Qaidamford cattle, cattle-yak, and Angus cattle to guide further improvement of Qaidamford cattle meat quality. RNA-seq sequencing has greatly facilitated the efficiency of identifying differentially expressed genes and this technology has been widely applied in cattle. In bovine embryo transcriptome analysis, RNA-seq technology was first applied in cattle to obtain digital gene expression [8]. In the Wagyu and Holstein subcutaneous fat transcriptome analysis, RNA-seq results revealed 662 differentially expressed genes, with some involved in adipogenesis and lipid metabolism [9]. Recently, comparative transcriptome analysis revealed that Angus ×  Qinchuan cattle (AQF) have improved performance characteristics compared to Qinchuan cattle (QCF) [10]. These studies provided the theoretical basis for this project.

In this study, our purpose was to screen differentially expressed genes in subcutaneous fat tissues of Qaidamford cattle, cattle-yak, and Angus cattle by RNA-seq. Gene ontology (GO) enrichment analysis revealed the involvement of some differentially expressed genes in fatty acid biosynthesis, lipid metabolism, and fatty acid catabolic biological processes. KEGG pathway analysis indicated that some differentially expressed genes participate in signal pathways, such as extracellular matrix (ECM)-receptor interaction and the PI3K-Akt signal pathway, which are important for adipose tissue development. In addition, real-time quantitative polymerase chain reaction (RT-qPCR) was used to verify the accuracy of RNA sequencing data. Together, these data can enable genetic improvement of hybrid cattle.

## 2. Materials and Methods

### 2.1. Animals and Adipose Tissue Sample

Qaidamford cattle and cattle-yak were used in this study. Three biological replicates were tested for each breed. The animals were slaughtered at about 24 months of age. Subcutaneous adipose tissue samples of each animal were collected from backfat and quickly transferred to liquid nitrogen. Sequencing data for Angus cattle (from five animals) were downloaded from the NCBI database (http://www.ncbi.nlm.nih.gov/Traces/study/?acc=SRP070110). Animal care and study protocols were approved by the Animal Care Commission of the College of Veterinary Medicine, Northwest A&F University.

### 2.2. RNA Extraction and RNA Sequencing

Samples of cattle subcutaneous fat tissue were subjected to RNA extraction. Total RNA was extracted using TRIzol (Takara) and treated with DNase I to remove the remaining DNA according to the manufacturer’s instructions. The purified RNA was then used for RNA sequencing. First, the mRNA was enriched using magnetic beads with Oligo (dT). Second, the mRNA was fragmented into short segments with fragmentation reagents, which was used for cDNA synthesis with random hexamers. Then, buffer, deoxyribonucleotide triphosphate(dNTPs), and DNA polymerase I were added to synthesize the second strand cDNA. Finally, the products were purified, subjected to sticky end repair, and ligated through the 3′-end plus the base “A” to a linker. The obtained fragments were subjected to size selection and PCR amplification enrichment. After qualifying with an Agilent 2100 Bioanalyzer (Davis, CA, USA) and the Real-Time PCR System (StepOnePlus; Applied Biosystems, Waltham, MA, USA), the constructed library was sequenced using the Illumina (San Diego, CA, USA) Sequencing Platform.

### 2.3. Sequencing Data Analysis

The original sequencing data, including low-quality reads, was filtered using Trimmomatic before proceeding to the next step [11]. After filtering, we aligned these high-quality reads to the genome using software *HISAT* (Johns Hopkins University, Baltimore, MD, USA) [12]. The gene expression level was the transcript abundance. Differential expression analysis was performed to identify differentially expressed genes among different samples and to perform deeper functional mining of differentially expressed genes. EdgeR was used to normalize the data and extract differentially expressed genes (DEGs) with FDR < 0.01. The data was uploaded to NCBI database and BioProject ID: PRJNA343359 (http://www.ncbi.nlm.nih.gov/bioproject/343359).

### 2.4. GO Enrichment and KEGG Pathway Analysis

GO enrichment analysis was used to classify the DEGs based on the specific biological functions [13]. Genes with different biological functions interact with each other to achieve the final function or phenotype. Pathway analysis can help characterize the biological function of genes. KEGG is the main public database of gene pathway, and can identify metabolic pathways or signal transduction pathways that are significantly enriched in differentially expressed genes as compared to the background of the entire genome.

### 2.5. Real-Time Quantitative PCR Analysis

To verify the RNA sequencing data, RT-qPCR was used to detect differential gene expression. Total RNA samples isolated from adipose tissue were reverse transcribed into cDNA using a PrimeScript™ RT reagent Kit (TaKaRa, Kyoto, Japan) with gDNA Eraser to remove genomic DNA. RT-qPCR analysis was performed in triplicate using a SYBR green kit (Genestar, Beijing, China) on an ABI StepOnePlus Real-Time PCR System. The expression levels of the selected differential genes were normalized against the expression level of the reference genes, β-actin and GAPDH. The reaction conditions were as follows: 95 °C for 10 min, 40 cycles of 95 °C for 15 s, 60 °C for 60 s. The relative expression levels of genes were calculated by the2^−ΔΔCt^ algorithm. The primers of all tested genes are shown in Table S1.

### 2.6. Statistical Analysis

Results are presented as the mean values ± standard error of the measurement (SEM). The significance of the difference of mRNA expression level (RT-qPCR data) between two groups was evaluated using two-tailed Student’s *t*-test with SPSS 19.0 statistical software. *p* value < 0.05 was considered statistically significant.

## 3. Results

### 3.1. Characterization of Bovine Adipose Tissue Transcriptome Sequencing Data

**Figure 1:**
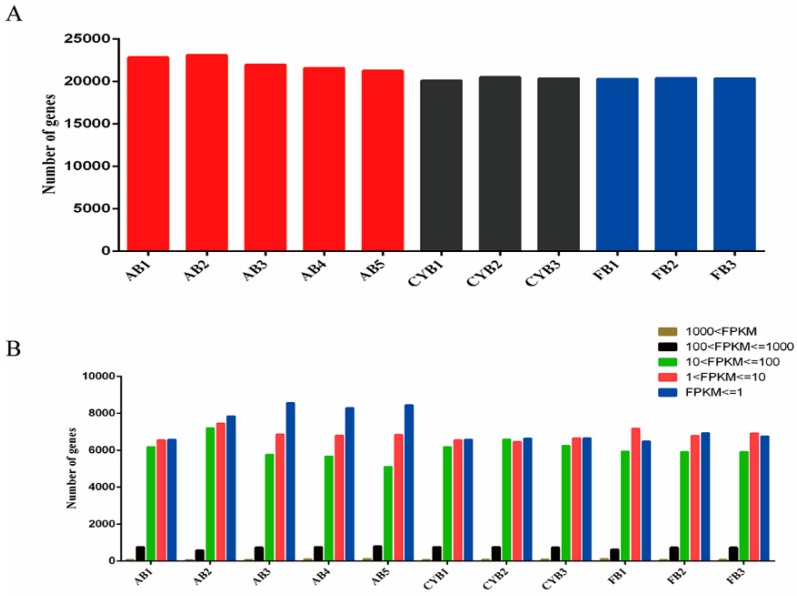
Characterization of bovine adipose tissue transcriptome sequencing data. (**A**) The total number of genes for each sample was calculated after counting the reads mapped in each gene. AB: Angus backfat; CYB: Cattle-yak backfat; FB: Qaidamford cattle backfat. (**B**) Statistics of the number of genes in each expression interval (fragments per kilobase of exon model per million mapped fragments, FPKM) in each cattle adipose tissue.

RNA-seq data were obtained for adipose tissue samples of cattle-yak (*N* = 3), Qaidamford cattle (*N* = 3), and Angus cattle (*N* = 5). The reads were mapped against the cattle reference genome (UMD_3.1.1) [14] (https://bovinegenome.elsiklab.missouri.edu/). The number of total raw reads and the percentage of clean reads of each sample were determined and are listed in (Table S2). After filtering, the total number of clean reads ranged from 42,023,896 to 46,456,047, with mapped reads as a percentage of the total ranging from 76.56% to 88.86%. For all samples, at least 72.95% of the reads uniquely mapped to the reference genome. Counting the reads mapped in each gene, the total number of mapped genes for each sample was calculated and is presented in Figure 1A. The distribution of mapped reads was similar for all samples. The number of genes in each expression interval (fragments per kilobase of exon model per million mapped fragments, FPKM) in each cattle adipose tissue sample was shown in (Figure 1B). These findings indicated good data quality that were suitable for subsequent research analysis.

### 3.2. Identification of Differentially Expressed Genes (DEGs)

**Figure 2:**
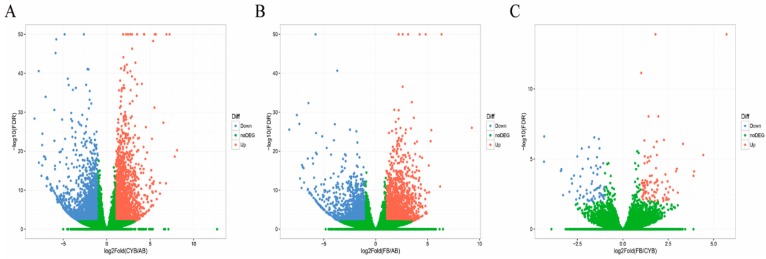
Volcanic maps represent the number of differentially expressed genes in adipose tissue of different cattle breeds. (**A**) Compared with the adipose tissue of Angus, differentially expressed genes were identified in the adipose tissue of cattle-yak. Up: Upregulated genes; Down: Downregulated genes; no DEG: No differentially expressed genes. (**B**) Compared with the adipose tissue of Angus, the number of differentially expressed genes were identified in the adipose tissue of Qaidamford cattle. (**C**) Compared with the adipose tissue of cattle-yak, the number of differentially expressed genes were identified in the adipose tissue of Qaidamford cattle.

We next investigated the differences in gene expression data for genes of adipose tissues between different cattle breeds. The analysis was performed as in previous studies. Reads were first normalized as FPKM [15]. Compared to Angus cattle, 4167 differentially expressed genes were identified in cattle-yak, of which 2144 genes were upregulated and 2023 genes were downregulated (Figure 2A, Supplementary Excel S1). A total of 3269 differentially expressed genes were identified between Angus cattle and Qaidamford cattle, including 1769 upregulated genes and 1500 downregulated genes (Figure 2B, Supplementary Excel S2). However, there were fewer differentially expressed genes identified between cattle-yak and Qaidamford cattle. Compared to cattle-yak, only 154 differentially expressed genes were identified, including 89 upregulated genes and 65 downregulated genes (Figure 2C, Supplementary Excel S3). These data suggest that there are some differences between hybrid cattle and their ancestors, which may guide the screening conditions for the subsequent breeding.

### 3.3. GO Enrichment and KEGG Pathway Analysis of Related DEGs

**Figure 3:**
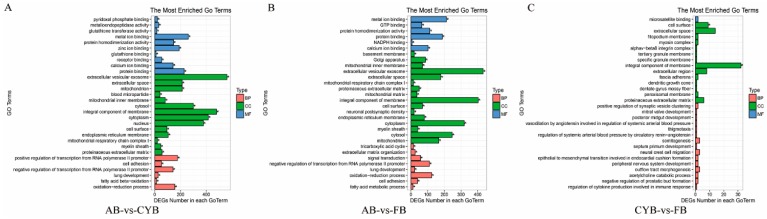
Gene ontology (GO) enrichment analysis of related differentially expressed genes (DEGs). (**A**) GO enrichment analysis of differentially expressed genes of cattle-yak compared with Angus. BP: Biological process; CC: Cell components; MF: Molecular function. (**B**) GO enrichment analysis of differentially expressed genes of Qaidamford cattle compared with Angus. (**C**) GO enrichment analysis of differentially expressed genes of Qaidamford cattle compared with cattle-yak.

To investigate the relationship between differentially expressed genes and adipose tissue formation, we used GO analysis using David software (https://david.ncifcrf.gov/) to analyze the differentially expressed genes. We found that some differentially expressed genes were enriched in fatty acid beta-oxidation biological process in cattle-yak adipose tissue compared to Angus cattle adipose tissue (Figure 3A). Further analysis revealed that all 14 differentially expressed genes were downregulated, including key genes influencing fat deposition such as ADIPOQ. Compared with Angus cattle, the differentially expressed genes in Qaidamford cattle were significantly enriched for the process of fatty acid metabolism (Figure 3B). However, the differentially expressed genes between cattle-yak and Qaidamford cattle showed no significant enrichment in biological processes (Figure 3C), which might suggest very little difference in fat content.

**Figure 4:**
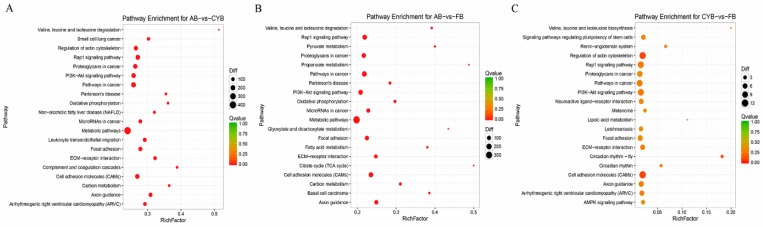
KEGG pathway analysis of related DEGs. (**A**) Cattle-yak compared to the Angus, the differentially expressed genes were subjected to pathway enrichment analysis. (**B**) Qaidamford cattle compared to Angus, the differentially expressed genes were subjected to pathway enrichment analysis. (**C**) Qaidamford cattle compared to the cattle-yak, the differentially expressed genes were performed to pathway enrichment analysis.

To further study the differentially expressed genes involved in the molecular pathway of adipose tissue development, the KEGG database was used to identify key candidate genes. Compared to Angus cattle, 3079 out of 4167 differentially expressed genes of cattle-yak were enriched in 300 pathways (Supplementary Excel S4). Some differentially expressed genes were enriched in important pathways like PI3K-Akt signaling (Figure 4A). A total of 2439 genes out of 3269 differentially expressed genes in Qaidamford cattle, relative to Angus cattle, were enriched in 299 pathways (Supplementary Excel S5). Some differentially expressed genes were also associated with the PI3K-Akt signal pathways (Figure 4B). Compared to the levels in cattle-yak, there were 154 differentially expressed genes of Qaidamford cattle. Of these 154, 114 were involved in 143 pathways (Supplementary Excel S4). Some differentially expressed genes were enriched in the PI3K-Akt signaling pathway but were not particularly significant (Figure 4C).

### 3.4. DEGs Were Validated Using RT-qPCR

**Figure 5:**
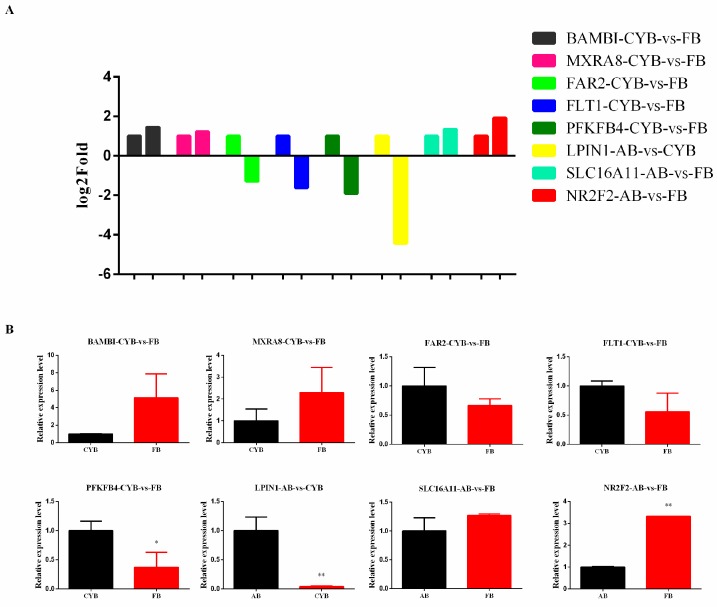
Verification of the differentially expressed genes by real-time quantitative polymerase chain reaction (RT-qPCR). (**A**) The RNA sequencing results revealed differentially expressed genes in adipose tissue of different cattle breeds. (**B**) Differentially expressed genes were confirmed by RT-qPCR. Data are represented as mean values ± SEM. *n* = 3. * *p* < 0.05, ** *p* < 0.01.

To confirm that the mRNA expression levels of the differentially expressed genes were consistent with the levels shown in these sequencing analysis data, we selected several genes involved in lipid-related processes for verification. RT-qPCR was performed to confirm the transcriptome analysis differences, as shown in Figure 5A. RT-qPCR was used to detect the expression of *SLC16A11*, *NR2F2*, *BAMBI*, *MXRA8*, *FAR2*, *FLT1*, *PFKFB4,* and *LPIN1* between different groups (Figure 5B). The gene expression levels showed that the RT-qPCR results were consistent with the RNA-seq analysis, suggesting the reliability of RNA-seq results.

## 4. Discussion

The aim of this study was to identify differentially expression genes related to adipose tissue formation indifferent cattle breeds. We used RNA-Seq technology to analyze transcriptome differences in the subcutaneous tissue of Qaidamford cattle, cattle-Yak and Angus cattle. The comparison of different bovine adipose tissue transcripts levels revealed some differentially expressed genes that are likely involved in the biological processes of fat formation, and some differentially expressed genes that participate in vital signaling pathways involved in adipose tissue development. Overall, understanding difference in gene expression in adipose tissue in Qaidamford cattle, cattle-yak, and Angus cattle can lead to future breed improvement.

RNA-seq is a high-throughput sequencing technology that is a powerful way to obtain large quantities of transcriptome data from many organisms, tissue types, and cell contexts, making it a convenient and efficient way to study gene expression on a genome-wide scale [16,17]. This method has been widely used in chicken [18], ducks [19], pigs [20], and cattle [8]. Especially in the study of adipose tissue and adipocytes, RNA-seq was used to identify differentially expressed RNAs including mRNA [21], miRNAs [22], and LncRNAs [23]. In this work, we applied RNA-seq to analyze the adipose tissue transcriptome in Qaidamford cattle, cattle-yak, and Angus cattle, and identified 4167 differentially expressed genes (Angus vs. cattle-yak), 3269 differentially expressed genes (Angus vs. Qaidamford cattle), and 154 differentially expressed genes (cattle-yak vs. Qaidamford cattle). Many differentially expressed genes were identified in the comparison of hybrid cattle and Angus cattle, suggesting significant differences in the adipose tissue. However, fewer differentially expressed genes were identified in the comparison of cattle-yak and Qaidamford cattle adipose tissue, suggesting little difference in adipose tissue.

GO analysis of these transcripts showed significant enrichment in GO categories related to fatty acid beta-oxidation and fatty acid metabolic process. Compared to Angus cattle, 14 differential expression transcripts were involved in fatty acid beta-oxidation of cattle-yak adipose tissue. These genes are closely related to fat deposition, and include adiponectin (ADIPOQ). Recent studies have shown that ADIPOQ plays a vital role in adipocyte development [24]. Twelve differentially expressed transcripts were related to fatty acid metabolic process in Qaidamford cattle adipose tissue. However, the number of difference genes between Qaidamford cattle and cattle-yak were not significant, suggesting little difference in adipose tissue.

KEGG pathway annotation of these genes revealed that ECM-receptor interaction and PI3K-Akt signaling were enriched pathways in the adipose tissues of the three breeds of cattle. ECM-receptor interaction was previously investigated in depot-specific adipogenesis in cattle [25]. The PI3K-Akt signaling pathway is a classic insulin signaling pathway [26], and is involved in proliferation, differentiation, apoptosis, and glucose transport. Moreover, the PI3K-Akt pathway may also regulate adipogenesis [27,28]. The detailed function and regulatory mechanisms of the differentially expressed genes involved in these pathways should be carefully elucidated in subsequent studies.

To ensure the accuracy and reliability of our sequencing results, it was necessary to use RT-qPCR. In this study, we prioritized the validation of genes differentially expressed in processes associated with adipose development. BAMBI has been investigated to negative regulate adipogenesis [29]. SLC16A11 might have a role in hepatic lipid metabolism and its genetic variation might result in diabetes risk [30,31]. NR2F2 (Coup-TF2) is a member of the steroid/thyroid hormone receptor family, which has been reported to negatively regulate adipogenesis [32]. The meat analysis experiment showed that the fat content of cattle was higher than that of hybrid cattle, which might be related to the expression of NR2F2 gene. Hence, the relationship of NR2F2 expression with fat content in cattle needs further study and discussion. Studies have shown that MXRA8 expression level is associated with increased marbling in beef cattle [33]. In addition, LPIN1 is a candidate gene for fat deposition in pigs [34]. These differentially expressed genes may have important roles in adipogenesis, and the regulatory functions of these candidate genes should be investigated in future studies.

## 5. Conclusions

The RNA-seq analysis identified differentially expressed genes in adipose tissue of Angus cattle, cattle-yak, and Qaidamford cattle. Some differentially expressed genes are involved in important signaling pathways, such as the PI3K-Akt and ECM-receptor interaction pathways. These transcriptome analysis data should allow subsequent bovine cross-breeding for improved meat quality.

## Supplementary Materials

The following are available online at https://www.mdpi.com/2076-2615/9/12/1077/s1, Table S1. Specific primers used for RT-qPCR. Table S2. Summary of sequence read alignments to the reference genome. Supplementary Excel S1. AB-vs.-CYB. DESeq. difffilter. Supplementary Excel S2. FB-vs.-AB. DESeq. difffilter. Supplementary Excel S3. CYB-vs.-FB. DESeq. difffilter. Supplementary Excel S4. Pathway enrichment analysis of AB-vs.-CYB. Supplementary Excel S5. Pathway enrichment analysis of AB-vs.-FB. Supplementary Excel S6. Pathway enrichment analysis of CYB-vs.-FB.
