# Supplementary material for: RNA-Seq Analysis Identifies Differentially Expressed Genes in Subcutaneous Adipose Tissue in Qaidaford Cattle, Cattle-Yak, and Angus Cattle
Source: Animals (Basel). 2019 Dec 3;9(12):1077. doi: 10.3390/ani9121077 (PMC6941056; doi:10.3390/ani9121077)
Supplement: Supplementary file 1 [file animals-09-01077-s001.zip › Supplementary Files20191121/Table S1.docx]

| Gene name | Forward | Reverse |
| --- | --- | --- |
| SLC16A11 | TCCCCTGTCAGGCTTCCTAA | CTTGGGCAGCCCCAAATAGA |
| NR2F2 | TGGAAAGCTTTTGCTTCGCC | GGCCAGTTAAAACTGCTGCC |
| BAMBI | CGCCACTCCAGCTACATCTT | GTAACCAGTAGCCACGCAGT |
| MXRA8 | TTCTCGCTGCTTATCGACCC | GTGACCCTCAGGTGGAAGAC |
| FAR2 | AGCCCAGGATGACAAAGCTC | GGACTCAGCTCGGACATCAG |
| FLT1 | AGGACCTGAAGCTGTCTTGC | GTTGCGTGGTCTGGTTGTTC |
| PFKFB4 | ACCCAGAACCCCCTGAAGAA | AGCCCCACCATGACAATGAG |
| LPIN1 | CTTCAGTTTCCCGACCGTCA | CATCTTGTGTGGCGAGGACT |
| GAPDH | CACTGAGGACCAGGTTGTCT | TGTCGTACCAGGAAATGAGC |

**Supplementary Table 1. Specific primers used for RT-qPCR**
