# Supplementary material for: RNA-Seq Analysis Identifies Differentially Expressed Genes in Subcutaneous Adipose Tissue in Qaidaford Cattle, Cattle-Yak, and Angus Cattle
Source: Animals (Basel). 2019 Dec 3;9(12):1077. doi: 10.3390/ani9121077 (PMC6941056; doi:10.3390/ani9121077)
Supplement: Supplementary file 1 [file animals-09-01077-s001.zip › Supplementary Files20191121/Table S2.docx]

| **Type** | **AB1** | **AB2** | **AB3** | **AB4** | **AB5** | **CYB1** | **CYB2** | **CYB3** | **FB1** | **FB2** | **FB3** |
| --- | --- | --- | --- | --- | --- | --- | --- | --- | --- | --- | --- |
| Total Raw Reads | 46396291 | 46231339 | 47326950 | 48314491 | 46500019 | 42337246 | 42335710 | 42336508 | 42337312 | 42335646 | 42336096 |
| Total Clean Reads | 43973586 | 45020680 | 46317561 | 46456047 | 45521013 | 42049938 | 42104050 | 42110920 | 42092658 | 42023896 | 42030662 |
| Total Clean Reads Ratio(%) | 94.78 | 97.38 | 97.87 | 96.15 | 97.89 | 99.32 | 99.45 | 99.47 | 99.42 | 99.26 | 99.28 |
| Total mapped reads(%) | 80.09 | 88.86 | 82.98 | 76.56 | 79.15 | 82.17 | 84.16 | 81.71 | 86.76 | 82.46 | 86.43 |

**Supplementary Table 2. Summary of sequence read alignments to the reference genome**
